# Sequencing by ligation variation with endonuclease V digestion and deoxyinosine-containing query oligonucleotides

**DOI:** 10.1186/1471-2164-12-598

**Published:** 2011-12-12

**Authors:** Antoine Ho, Maurice Murphy, Susan Wilson, Susan R Atlas, Jeremy S Edwards

**Affiliations:** 1 Department of Molecular Genetics and Microbiology, University of New Mexico. Albuquerque, New Mexico, USA; 2 Cancer Center, University of New Mexico. Albuquerque, New Mexico, USA; 3 Center for Advanced Research Computing, University of New Mexico. Albuquerque, New Mexico, USA; 4 Department of Physics and Astronomy, University of New Mexico. Albuquerque, New Mexico, USA; 5 Department of Chemical and Nuclear Engineering, University of New Mexico. Albuquerque, New Mexico, USA

## Abstract

**Background:**

Sequencing-by-ligation (SBL) is one of several next-generation sequencing methods that has been developed for massive sequencing of DNA immobilized on arrayed beads (or other clonal amplicons). SBL has the advantage of being easy to implement and accessible to all because it can be performed with off-the-shelf reagents. However, SBL has the limitation of very short read lengths.

**Results:**

To overcome the read length limitation, research groups have developed complex library preparation processes, which can be time-consuming, difficult, and result in low complexity libraries. Herein we describe a variation on traditional SBL protocols that extends the number of sequential bases that can be sequenced by using Endonuclease V to nick a query primer, thus leaving a ligatable end extended into the unknown sequence for further SBL cycles. To demonstrate the protocol, we constructed a known DNA sequence and utilized our SBL variation, *cyclic SBL *(cSBL), to resequence this region. Using our method, we were able to read thirteen contiguous bases in the 3' - 5' direction.

**Conclusions:**

Combining this read length with sequencing in the 5' - 3' direction would allow a read length of over twenty bases on a single tage. Implementing mate-paired tags and this SBL variation could enable > 95% coverage of the genome.

## Background

Following the completion of the human genome project it is anticipated that genome sequencing of an individual will be an aspect of routine treatment for a number of diseases and illnesses, truly ushering in the era of personalized medicine. However, the reality of implementing genome sequencing as a medical tool depends on the cost of sequencing technology [1]. The price tag on the human genome project was $2.7 billion, requiring the labor of hundreds of scientists, and a decade's worth of time [2]. By contrast, sequencing and analyzing a human genome can now be performed for under $50,000 in about four months' time with the labor of a few individuals [3-5]. This advance was made possible by progressing from traditional Sanger sequencing methods to so-called "next-generation" methods that focused on miniaturization of the sequencing reactions, massive parallelization of data acquisition, and computational analysis. This not only resulted in increased sequencing speeds, but also significantly reduced the cost of genome sequencing [6]. However, in order to expand the use of genomic analysis to the clinic, price, quality, and speed must all be advanced further [7-14].

Sanger sequencing remains the gold standard today for accurate DNA sequencing. Sanger sequencing can reach read lengths of up to roughly 1,000 base pairs, dwarfing most current next-generation methods that average fewer than 100 base pairs [15]. What next-generation methods accomplish is massive parallelization, resulting in throughputs that are orders of magnitude greater than Sanger sequencing. However, the throughput gains come at a cost of a reduced read length [1,16,17]. Therefore, Sanger sequencing will remain an essential laboratory tool for years to come; although, for the purposes of large sequencing projects (i.e. whole genome sequencing, exome sequencing, RNAseq, ChipSeq, etc.), next-generation methods are the new standard [18].

There are multiple sequencing methods that are utilized in next-generation methods. The two most common can be broadly categorized as Sequencing By Synthesis (SBS) [19-21] and Sequencing By Ligation (SBL) [22,23]. SBS is a method of sequencing which utilizes a DNA Polymerase enzyme to incorporate a single fluorescently labeled nucleotide that contains a reversible terminator. This allows a period of data acquisition before removal of the fluorophore, reversal of the terminator, and continuation of sequencing [24]. Additionally, there are single molecule and real-time SBS approaches [25,26], which, as their names imply, are performed without template amplification and sequenced in real-time using some indicator of nucleotide incorporation. In the present work, we have focused on increasing the read length of SBL.

SBL is a straightforward enzymatic method of sequencing DNA. SBL uses known, universal sequences that flank an unknown genomic tag as anchor primer sites [22]. An *anchor primer *is hybridized to one of these known regions, and a ligatable end (3' or 5' depending on the direction of desired sequencing) is available. An oligo, called a *query primer*, is then ligated to the end of the anchor primer. The query primer is a mix of oligos that are degenerate for all positions except a single position that is being sequenced, which allows the sequencing of a single position based on the design of the query primer. After sequencing a single position, the query primer and anchor primer are stripped from the DNA template, effectively resetting the sequencing. The process begins again, sequencing a different position by using a different query primer, and repeating until the entire sequence of the tag has been determined [23]. Increased read length can be accomplished either by increasing the distance SBL can be performed in a single direction, or by incorporating additional universal regions for more anchor primer sites [5,22].

**Figure 1:**
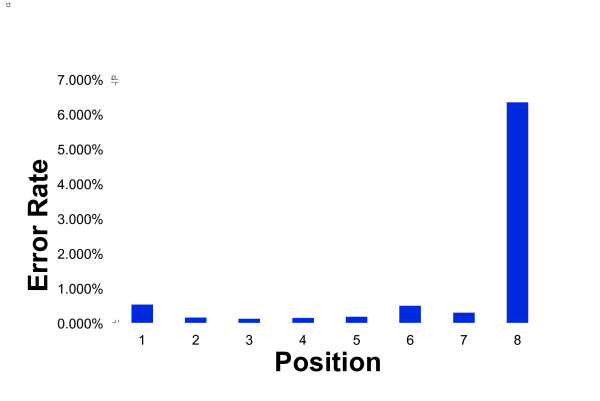
**Errors and Error Rate versus Position**. Unpublished results in a traditional SBL sequencing run. These reads are separated into two parts, A and B, and are designed either M or P for Minus or Plus, away or towards the site of attachment on the bead. These AM and AP reads are obtained using different hybridized primers. Error and error rates, when not using a cyclic or digestion method, results in loss of specificity the further away a base is from the site of ligation.

Currently, the number of sequential bases that SBL-based approaches can sequence is limited by loss of specificity of base pair hybridization at any distance away from the site of ligation. Errors in the first six base pairs adjacent to the site of ligation are rare due to the destabilizing effect of mismatches. However, at a distance of about seven base pairs, the specificity of the SBL reaction is reduced (Figure 1). Therefore it is not possible to simply use longer and longer query primers in order to increase SBL read lengths [27].

In this manuscript, we describe a variation on SBL that utilizes a deoxyinosine in the query primer that can be cleaved by Endonuclease V [28] to increase the read length through successive cycles, which we refer to as *cyclic SBL *or cSBL. Our approach is conceptually similar to the ABI SOLiD method of SBL, which uses a chemical cleavage of the query primer to get extensions of read lengths. However, in contrast, our method utilizes an enzymatic cleavage using completely off-the-shelf reagents. Deoxyinosine is a universal base [29] that is recognized by Endonuclease V, which cleaves between the 2^nd ^and 3^rd ^phosphodiester bond 3' from the deoxyinosine site [28]. Cyclic SBL is thus identical to standard SBL except that there is a deoxyinosine incorporated in the query primers that is used for cleavage. Therefore, after ligation of a query primer onto an anchor primer, one can use Endonuclease V to cleave off the end of the query primer. This cleavage results in a ligatable end with a portion of the query primer is still ligated to the anchor primer, effectively lengthening the anchor primer for an SBL reaction to increase the SBL read length. The cycles of ligation and Endonuclease V digestion can be repeated to further increase the read length. We have used this approach to extend the read length of SBL to thirteen base pairs in the 3' - 5' direction.

## Results

### Cyclic SBH

**Figure 2:**
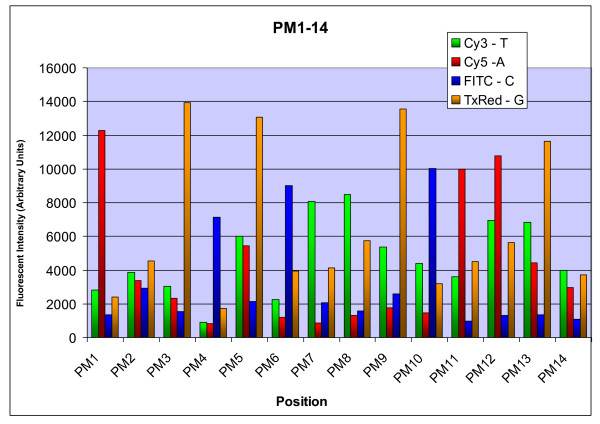
**Fluorescent Intensity plotted versus position for each channel, for the Test Template sequence**. Sequenced area is underlined. 5' TCT ATG GGC AGT CGG TGA TANGCG CTT GCA AGA GAA TGA GGA AAA CGA AGA 3'.

Three cycles of cSBL were performed, giving accurate signal for the first 13 positions of the Test Template. There was a slight increase in non-specific signal with each cycle, but the third cycle still had clearly correct signal with an acceptable signal to noise ratio (Figure 2).

We were unable to sequence the 14^th ^position and beyond using the cSBL strategy. In order to determine the possible cause of this, we performed a series of tests to explore whether the template DNA had been digested by the Endonuclease V treatment, since this seemed the most likely problem. After the beads had undergone cSBL and stripping of the sequencing strand of DNA, we hybridized a fluorescent probe to the 3' end of the DNA loaded onto the beads and confirmed that the Test Template was still present on the bead.

We also ruled out the issue of secondary structure causing the 3' end of our Test Template to become inaccessible. We performed folding calculations using IDTDNA's Oligo Analyzer software (29) when constructing our Test Template specifically in order to avoid secondary-structure problems. Calculations for melting temperatures (T_M_) of secondary structures were performed assuming 50 mM Na^+ ^and 10 mM Mg^++^. This simulated the highest folding T_M _at 31.5 degrees, and the fold as modeled by the software was not located near the 14^th ^base pair.

We additionally performed ligation at 50°C using Taq DNA Ligase (NEB), which has a higher optimal temperature, but could not obtain the 14^th ^position or further. We have been unsuccessful in identifying a definitive reason for the observed sequencing limit of 13 continuous bases. However, based on the results from Figure 2, our cSBL strategy does consistently provide at least thirteen base-pair reads in the 3' - 5' direction, and can easily reach twenty-three bases with the addition of a flanking anchor primer site and 5' - 3' sequencing of 10 bases.

### Read Length Versus Genome Coverage

To demonstrate the feasibility of a cSBL approach to genome sequencing and calculate gains in using cSBL over traditional SBL methods, we utilized the SawTooth resequencing code developed at the University of New Mexico (M. Murphy *et al*., to be submitted, 2011). Human genome coverage was simulated using mate-paired data ranging from twenty-six bases to (limit of traditional SBL) to forty bases (theoretical gain from cSBL implementation).

A set of simulated mate-paired tags, each separated by a range of 300-700 bases, was created, ranging in size from 13 paired tags to 20 paired tags. A sufficient number of tags were computationally generated to simulate 10 × coverage. The tags were all generated from chromosome 1, mapped back to the entire genome, and calculations of chromosome 1 coverage were performed. Mapping tags back to the whole genome, instead of just chromosome 1, provided a more realistic comparison to how human genome sequencing is typically performed [30,31]. Tags that mapped to multiple locations, whether in the entire human genome or chromosome 1, were discarded. A tag that *maps uniquely *or maps back to the reference genome in a single location provides useful data. If a tag maps uniquely to the reference sequence, the loci where it maps are said to be *covered *by that tag. For a given locus, the number of all such unique mappings when all tags are considered is called the *depth of coverage *for that locus. SAWTooth uses a general hash index, perhaps the fastest data retrieval structure. Although there are some limitations to general hash indexes, the nature of genomic data and the specialized task of mapping paired end reads to a reference genome, allows the use of hash indexes that circumvent these limitations.

**Figure 3:**
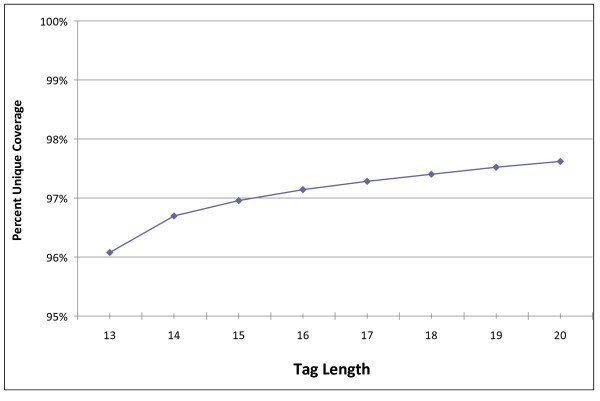
**Percent of sequenced regions on chromosome 1 covered by at least one unique mapping, as a function of tag length**.

**Figure 4:**
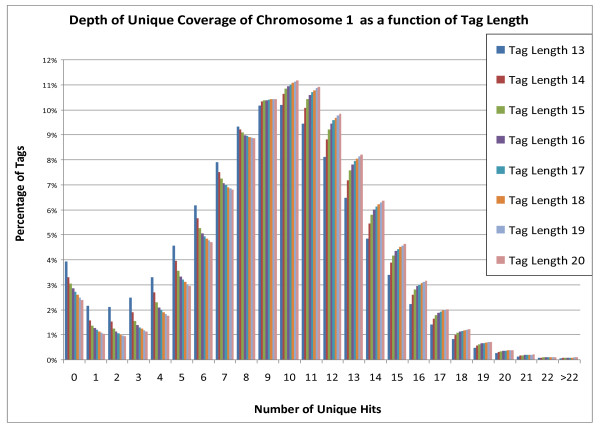
**Depth of unique coverage of sequenced regions on chromosome 1 at various tag lengths**.

**Figure 5:**
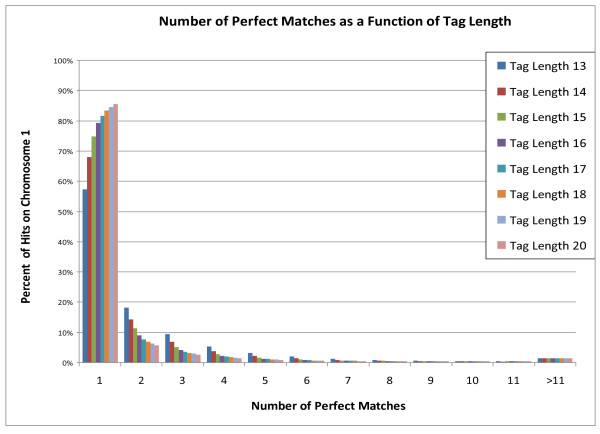
**Simulated percentage of reads and the number of perfect matches as a function of tag length**.

The SawTooth mapping analysis yielded the results summarized in Figures 3, 4, 5. Figure 3 shows raw coverage of chromosome 1 as a function of tag length. Increasing tag lengths from thirteen to twenty, or twenty-six to forty total bases while mate-paired, results in an increased coverage of chromosome 1 from 96% to 97.5%. Gains of coverage are significant when the read lengths are small, but suffer from diminishing returns as read length increases. Also, as expected, depth of coverage increases with tag length (Figure 4).

Next, we performed an analysis of how many times each tag mapped to the genome. One of the more significant benefits gained by increasing tag length from 13 to 20 bases is that far fewer tags must be discarded because they do not map uniquely (see Figure 5). At a tag length of 13 bases, only 57.2% of the tags are used, compared to 85.6% at a tag length of 20, thus effectively increasing throughput.

## Discussion

The cSBL protocol described here is a variation on traditional SBL that can increase the read lengths by increasing the number of contiguous bases sequenced. Implementation of the cSBL approach could potentially increase reads to twenty-three base pairs, or forty-six total base pairs with a mate-paired constructed library. In this manuscript, we performed the sequencing on a test DNA template rather than a genome library. however, we expect that any biases or mismatches in our cSBL will be exactly the same as general SBL. These issues include increased mismatches in specific positions of the query primer [32], or general drops in efficiency when dealing with A or T rich regions of the genome [27]. Additionally, our experiments were performed on beads suspended in solution rather than on beads immobilized on a surface. Therefore, to implement our sequencing strategy in a next generation sequencing platform, the methods would need to be optimized on immobilized beads.

Our cSBL strategy is not truly bi-directional. This is because Endonuclease V cuts in the 3' direction relative to the deoxyinosine position. Therefore, using Endonuclease V for cSBL in the 5' to 3' direction would result in the deoxyinosine remaining in the extended anchor primer. This would limit the number of cSBL cycles in the 5' to 3' direction to two, as attempts to go further will recognize the first incorporated deoxyinosine and limit the extended reads in the 5' to 3' direction.

## Conclusions

In summary, we have demonstrated that next-generation sequencing approaches applying the cSBL variation will be able to produce longer read lengths relative to standard SBL. Additionally, cSBL is compatible with and further increases the sequence gains from methods that incorporate additional anchor primer sites. Also, cSBL can complement traditional SBS approaches as cSBL can sequence in the 3' to 5' direction. This variation of traditional SBL approaches has useful applications in many next-generation sequencing methods that are in active use today.

## Methods

We have applied cSBL to sequence a known test DNA fragment (Test Template, see Table 1) immobilized on 1.0 um beads (MyOne Beads, Invitrogen) in solution. All DNA primers used were synthesized by Integrated DNA Technologies. The Test Template was constructed not to have significant secondary structure. The 5' end of the Test Template is modified with a dual biotin on the 5' end to couple to streptavidin-coated beads. The anchor primers (Anchor Primer, see Table 1) were designed to hybridize onto the 5' end of the Test Template, and provide a free 5' phosphate to ligate the query primers (Extension Primers, see Table 1). Multiple anchor primers that were identical except that each progressive primer was shorter by one nucleotide were used. The multiple anchor primers allowed multiple positions to be sequenced with the same set of query. In addition to the query primers, we used a Saturation Primer. The purpose of this was to fully saturate all available ligatable sites, therefore combating drops in signal efficiency and phasing in further cycles. In addition, a standard query primer that did not contain a deoxyinosine was used to sequence the 5^th ^and 10^th ^positions. The 10^th ^position was obtained following a single cycle of cSBL.

**Table 1:** Sequences of the Test Template, various Anchor Primers, and Query Primers.

Sequence Names	DNA Sequence
**Test Template**	5' (Dual Biotin) TCT ATG GGC AGT CGG TGA TAN GCG CTT GCA AGA GAA TGA GGA AAA CGA AGA 3'

**Anchor Primer**	5' (Phosphate) A TCA CCG ACT GCC CAT AGA 3'

**-1 Anchor Primer**	5' (Phosphate) TCA CCG ACT GCC CAT AGA 3'

**-2 Anchor Primer**	5' (Phosphate) CA CCG ACT GCC CAT AGA 3'

**-3 Anchor Primer**	5' (Phosphate) A CCG ACT GCC CAT AGA 3'

**ExSeq4 - A**	5' Cy3 - NNINNANNN 3'

**ExSeq4 - T**	5' TYE 665 (Cy5 Analog)- NNINNTNNN 3'

**ExSeq4 - C**	5' 6-FAM (FITC Analog)- NNINNCNNN 3'

**ExSeq4 - G**	5' TEX 615 (Texas Red Analog)- NNINNGNNN 3'

**Saturation Primer**	5' NNINNNNNN 3'

Deoxyinosine is indicated by "I." Degenerate bases represented by "N." Underlined bases are areas of anchor primer hybridization.

### Binding DNA to Beads

The dual-biotin on the test template was bound to the streptavidin-coated beads (MyOne Beads, Invitrogen, Carlsbad, CA). 30 uL of beads were washed three times in Bind and Wash Buffer (10 mM Tris-HCl, 1 mM EDTA, 2.0 M NaCL) and collected using a magnetic particle collector. The beads were then resuspended in 120 uL of BW Buffer and 1.2 uL of 1 mM Test Template sequence (10 uM final concentration) was added incubated at room temperature in a rotisserie for forty-five minutes. Finally, the beads were washed times and resuspended in 60 ul of Wash 1E (10 mM Tris, 50 mM KCl, 2 mM EDTA, and .01% Triton X-100).

### Hybridize Anchor Primer onto Template DNA

The beads were washed in Wash 1E (10 mM Tris, 50 mM KCl, 2 mM EDTA, and .01% Triton X-100), then washed once in a 1 × SSPE (150 mM NaCl, 10 mM NaH_2_PO_4_, and 1 mM EDTA pH 7.4). The beads were then resuspended in 150 uL 1 × SSPE with 2 uL of 1 mM anchor primer (13 uM final concentration). The solution was incubated at 50°C for 15 minutes and then cooled to room temperature for ten minutes. Lastly, the beads were washed in Wash 1E three times and immediately used in the Query Primer Ligation.

### Query Primer Ligation

The beads were collected in resuspended in the ligation buffer (66 mM Tris-HCL, 10 mM MgCl2, 1 mM dithiothreitol, 1 mM ATP, 7.5% Polyethylene glycol [PEG6000]), with a query primer concentration of 3 uM each, and T4 DNA Ligase (2 U/ml, NEB). The ligation reaction was incubated at 30°C for 45 minutes on a rotisserie. Following the reaction the beads were washed three times in Wash 1E and resuspended in Wash 1E. The fluorescent signal was verified using a fluorescent microscope.

### Microscope Fluorescent Calibration

**Figure 6:**
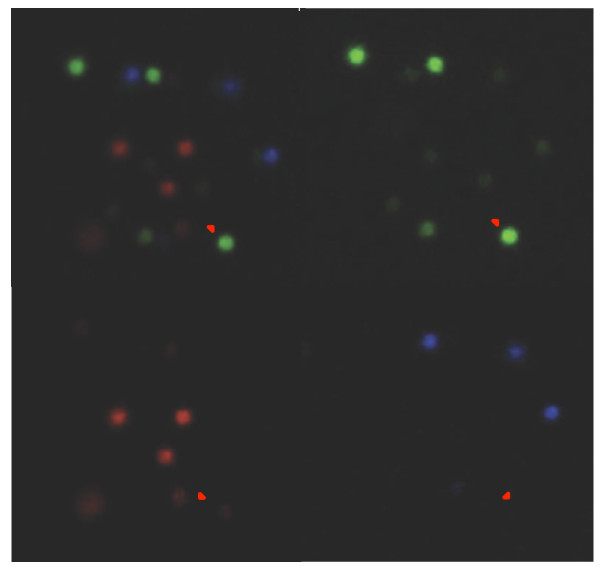
**An overlay of three channels of fluorescence**. In practice, there are four fluorescent channels, one corresponding to each base. Only three channels and the corresponding overlay are shown here for clarity. NIS-Elements Basic Research 3.0 (Nikon Instruments Inc, Melville, N.Y) software was used to generate this image and analyze the data. Pixel values are taken from beads in each channel to ascertain sequencing accuracy. The pixel values of brightness in each channel are used as a gauge of nucleotide identity. The pixel values of the brightest channel for a given bead and the values of other channels, provide the signal to noise ratio for comparison.

The exposure and gain for each fluorescent filter was adjusted with all positions present for each cycle. Camera settings were optimized each cycle of cSBL as signal dropped from one cycle to the next. The individual populations of beads were examined separately with the same settings, and then scored using NIS-Elements Basic Research imaging software (Nikon Instruments Inc, Melville, NY) (Figure 6).

### Pixel Intensity Evaluation as a Measure of Sequencing Accuracy

NIS-Elements Basic Research 3.0 (Nikon Instruments Inc, Melville, NY) was used to determine the pixel intensities in the Cy3, Cy5, FITC, and TxRed channels. Individual channel intensity values ranged from 1-16,383. One-hundred pixels were averaged in each channel and compared. This gave a metric for estimating sequencing accuracy, as the correct signal was known for each position.

### Saturation Ligation

A saturation step was performed to fully saturate all Anchor Primers sites not extended during the Query Primer ligation cycle. The ligation was performed in a 1 × T4 DNA Ligase Buffer, with a Saturation Primer concentration of 10 uM and T4 DNA Ligase (2 U/mL), at 30°C for forty-five minutes on a rotisserie.

### Endonuclease V Digestion

The beads were washed three times and resuspended in 1 × NEB4 (50 mM Potassium Acetate, 20 mM Tris-Acetate, 10 mM Magnesium Acetate, 1 mM Dithiothreitol) with 100 ug/mL BSA and Endonuclease V at a 2 U/mL concentration. The endonuclease V digestion was incubated at 37 degrees on a rotisserie for ten minutes. Removal of the fluorescence was confirmed visually using a fluorescent microscope. Specific digestion and negligible non-specific Endonuclease V digestion was confirmed by an overnight incubation with Endonuclease V with test-template bound beads. The overnight digestion resulted in no detectable non-specific endonuclease activity when gauged by hybridizing a probe to the distal region of the Test Template.

### Endonuclease V Deactivation

Following the Endonuclease V digestion, the beads were extensively washed to remove all Endonuclease V. Enzyme carry forward could cause phasing problems, therefore, a guanidine wash was also performed to inactivate residual enzyme. The bead solution was washed in a 3 M Guanidine solution at room temperature. Following the guanidine wash, the beads were washed three time and resuspended in Wash 1E.

### Cyclic Ligation

**Figure 7:**
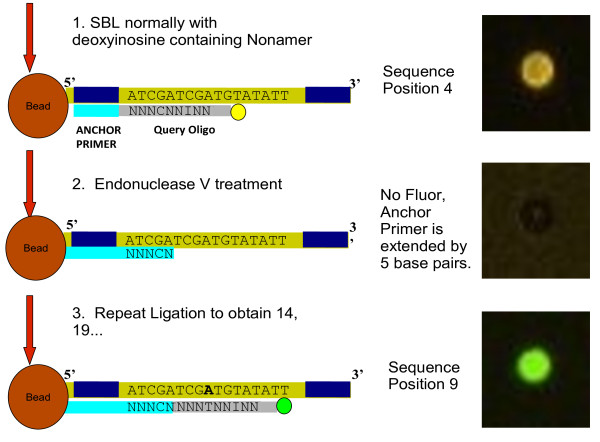
**Sequencing By Ligation with Endonuclease V Digestion**. 1) Sequencing the fourth base in the template tag, by using standard SBL with a Query Oligo that contains a Deoxyinosine (I). 2) Endonuclease V will recognize the Deoxyinosine and cleave the second phosphate bond towards the 3' end. The picture has white light background to make the bead visible as all fluorescence is ablated. 3) Repeat SBL to obtain the next positions.

After Endonuclease V deactivation, the template DNA has been sequenced in one position, but now the anchor primer is effectively lengthened. In traditional SBL, the sequencing strand would be stripped to repeat the sequencing process for a different position. With cSBL, the sequencing of additional bases is dependent upon the preservation of the hybridized sequencing strand of DNA. The process therefore begins again with query primer ligation, and is repeated until the signal to noise ratio is too low to effectively continue sequencing by SBL. At that point, the entire sequencing strand can be stripped and a different length anchor primer can be used to sequence different bases, as in traditional SBL (Figure 7).

## Authors' contributions

AH, MM, SW, SRA, and JE have all contributed to and participated in drafting this manuscript. All authors read and approved the final manuscript.
